# Chemotherapy-induced uridine diphosphate release promotes breast cancer metastasis through P2Y_6_ activation

**DOI:** 10.18632/oncotarget.8664

**Published:** 2016-04-09

**Authors:** Xiaobin Ma, Xinhua Pan, Yinglei Wei, Binhe Tan, Linli Yang, Hua Ren, Min Qian, Bing Du

**Affiliations:** ^1^ Shanghai Key Laboratory of Regulatory Biology, Institute of Biomedical Sciences and School of Life Sciences, East China Normal University, Shanghai, China

**Keywords:** UDP, P2Y_6_, MMP-9, metastasis, breast cancer

## Abstract

Although purinergic signaling is important in regulation of immune responses, the therapeutic potential of it in the tumor microenvironment is little defined. In this study, we demonstrate that UDP/P2Y_6_ signaling facilitates breast cancer metastasis both *in vitro* and *in vivo*. We found that P2Y_6_ is not only aberrantly expressed and mutated in most tumor types, but also highly correlated with poor prognosis in breast cancer patients. Furthermore, the migration and invasion of breast cancer cells was obviously increased by UDP and blocked by P2Y_6_ specific inhibitor MRS2578 and P2Y_6_ shRNA. Similar results was also found in breast cancer cell metastasis mouse model. Interestingly, the endogenous agonist UDP was released significantly by doxorubicin treated cells. In addition, the expression and enzyme activity of MMP-9 were both promoted by UDP and inhibited by MRS2578 or P2Y_6_ shRNA. Furthermore, UDP-induced cell invasion was blocked by an MMP-9 inhibitor. Mechanistically, the MAPKs and NF-κB signaling pathways, known to be involved in regulation of MMP-9 expression, were both activated by UDP. Taken together, our study reveals a relationship between extracellular danger signals and breast cancer metastasis, which suggests the potential therapeutic significance of UDP/P2Y_6_ signaling in cancer therapy.

## INTRODUCTION

Since the concept of purinergic signaling was first proposed in 1972 [1], there has been growing interest in performing functional and mechanistic studies into extracellular nucleotides in the context of both physiological and pathological processes. Among them, the therapeutic potential of purinergic signaling for the treatment of cancer has become popular [2]. For example, the anti-tumor activity of ATP was first described in pancreatic and colon cancer cells in 1983 [3], whereas ATP was also found to be released through pannexin-1 channels as a pro-metastatic signal that permits the survival of cancer cells [4]. Meanwhile, adenosine, derived from ATP hydrolysis, has been suggested to promote tumor growth [5] and metastasis through the A_2A_ receptor [6]. Previous studies have shown that extracellular UDP released from damaged or stressed cells is a danger signal that promotes innate immune responses [7, 8]. However, the function and mechanism of extracellular UDP signaling via the P2Y_6_ receptor in breast cancer metastasis remains unknown.

The repopulation, progression and even metastasis of tumors after anticancer therapies such as radiotherapy, chemotherapy and surgery is well-recognized [9]. Generally, both radiotherapy and chemotherapy are administered at low doses to avoid a severe toxic reaction in normal tissues. However, the surviving tumor cells can also recover from treatment, which might accelerate tumor progression. Interestingly, previous studies have shown that apoptotic or injured tumor cells release different nucleotides as a find-me signal for phagocytic clearance [10]. Furthermore, extracellular ATP and ADP can also bind to P2Y_1_ or P2Y_2_ receptors to facilitate microvascular metastatic cell survival and prostate cancer cell metastasis [11]. Although UDP is also released by damaged or stressed immune cells, the potential role of UDP and P2Y_6_ in the regulation of tumor formation and metastasis has been little studied. So, understanding whether UDP can be released in the tumor microenvironment after radiotherapy or chemotherapy and the potential of UDP\P2Y_6_ signaling in cancer cell metastasis will be important in developing treatments to address tumor repopulation, progression and metastasis.

In this study, we demonstrate that P2Y_6_ is aberrantly expressed and mutated in tumor tissues and is highly correlated with a poor prognosis in breast cancer patients. Extracellular UDP accumulates in doxorubicin-treated cells and facilitates breast cancer cell migration and invasion both *in vitro* and *in vivo*. Furthermore, extracellular UDP increases the expression and enzymatic activity of MMP-9 through ERK and NF-κB signaling by activating P2Y_6_. Therefore, we suggest that UDP/P2Y_6_ signaling not only functions as a danger signal in regulation of immune responses, but also as a prometastatic signal in breast cancer. This provides a rationale for the simultaneous use of these therapies in combination with P2Y_6_ inhibitors.

## RESULTS

### P2Y_6_ is highly correlated with breast cancer progression

**Figure 1:**
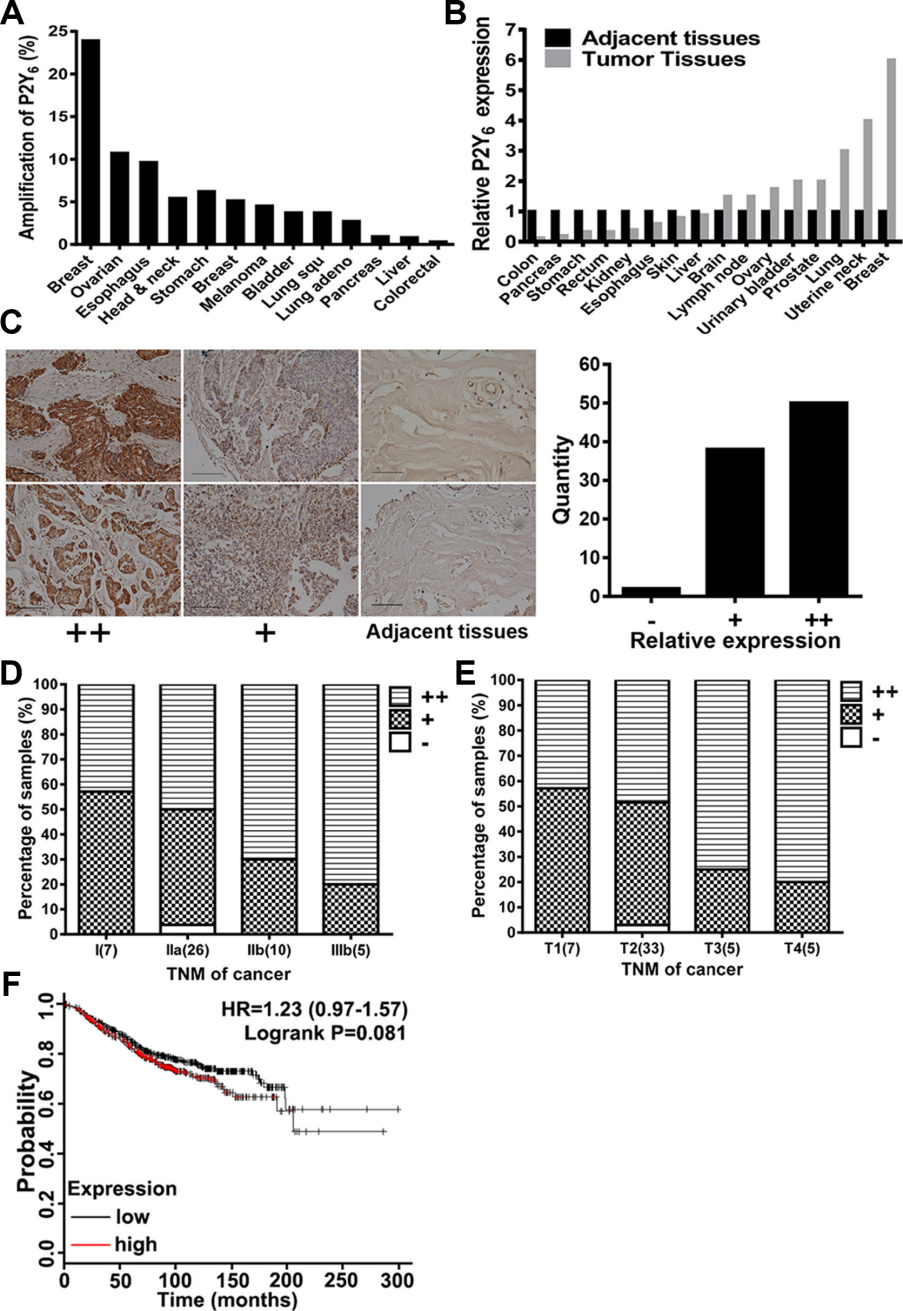
P2Y_6_ highly expressed in breast cancer tissues (**A**) The percentage of P2Y_6_ gene amplification (X-axis) in different cancer types (Y-axis) was analyzed in the TCGA database. (**B**) Relative expression of P2Y_6_ in different cancer types according to the adjacent tissues from clinical patients. (**C**) Relative expression of P2Y_6_ between breast cancer tissue and normal breast tissue (200×, *n* = 90). Expression of P2Y_6_ in breast cancer tissue and normal breast tissue from clinical patients was analyzed by IHC and distinguished into: high expression (++), low expression (+) and adjacent tissue (−). (**D**) Tumor stage was highly correlated with the expression of P2Y_6_. (**E**) Tumor size was highly correlated with expression of P2Y_6_. (**F**) Kaplan-Meier overall survival curves according to the P2Y_6_ mRNA level in breast cancer patients (lower vs. upper quartiles). P2Y_6_ expression in breast cancer tumors was negatively correlate with overall survival.

To illustrate the potential relationship between P2Y_6_ and cancer progression, The Cancer Genome Atlas (TCGA) database was analyzed. As shown in Figure 1A, up to 24% amplification of P2Y_6_ has been found in different cancer samples, especially in breast cancer xenografts. We then compared the expression level of P2Y_6_ in different clinical cancer samples with adjacent normal tissues by immunohistochemistry assay and found that the expression of P2Y_6_ in breast cancer tissues was highly elevated (Figure 1B). Furthermore, the expression level of P2Y_6_ was positively correlated with malignancy (Figure 1C), stage (Figure 1D) and TNM classification (Figure 1E) in human breast cancer. Accordingly, the expression of P2Y_6_ was also positively correlated with a poor prognosis in breast cancer patients (Figure 1F). Therefore, these data indicate that the expression and mutation of P2Y_6_ is dramatically increased in breast cancer tissues and suggest the potential role of P2Y_6_ in breast cancer formation and progression.

### Extracellular UDP facilitates breast cancer cell migration and invasion

**Figure 2:**
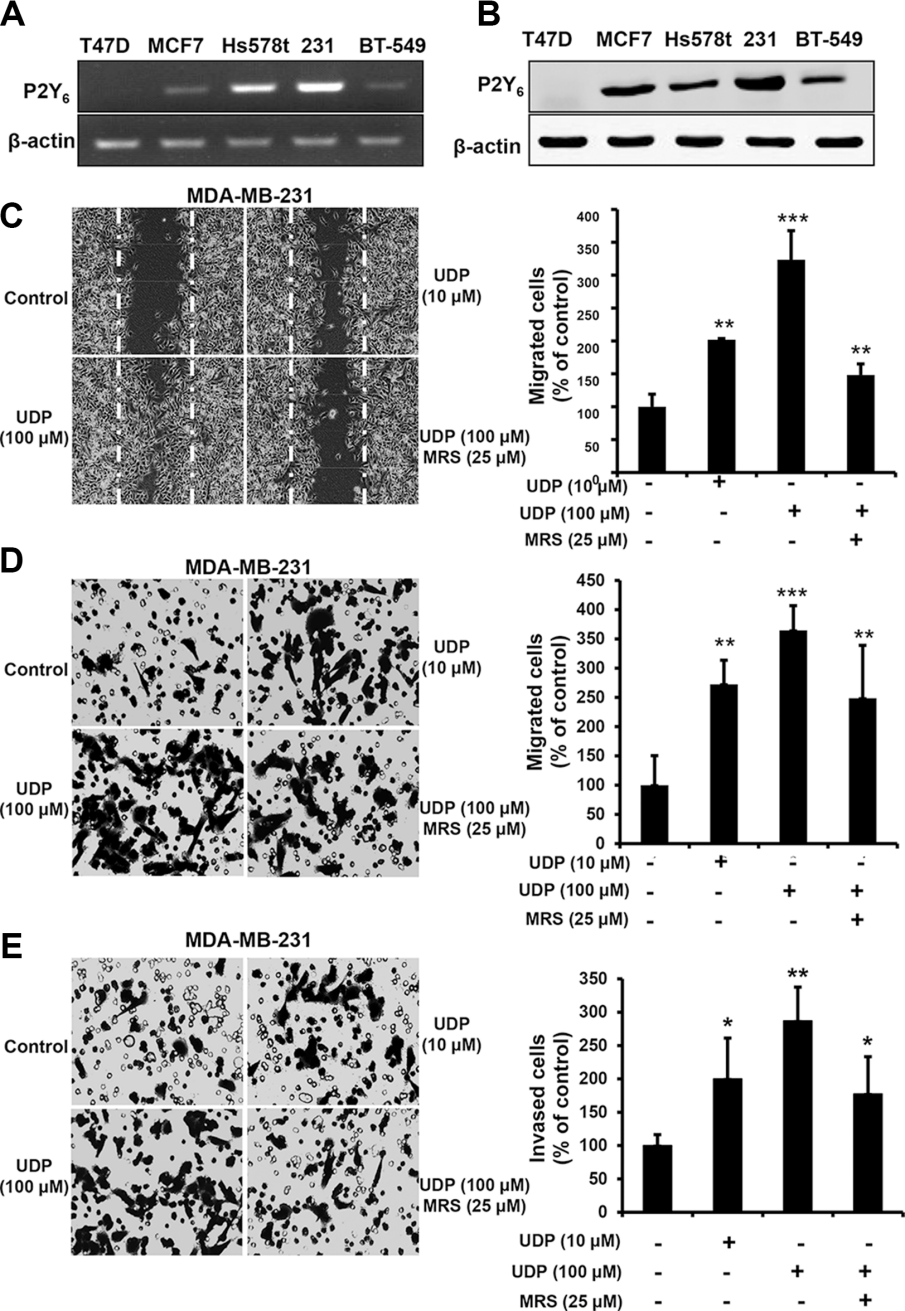
UDP promotes breast cancer cell metastasis (**A**) RT-PCR analyses of P2Y_6_ mRNA expression in different breast cancer cell lines. (**B**) Western blotting analyses of P2Y_6_ protein expression in different breast cancer cell lines. (**C**) MDA-MB-231 cells were scratched with a pipette tip and then treated with UDP or MRS2578 for 24 h. Then, the migrated cells were fixed and counted. Images were taken using a 20× objective. (**D**) MDA-MB-231 cells were pre-incubated with UDP or MRS2578 for 8 h in Transwell migration assays. Cells on the bottom side of the filter were fixed, stained and counted. The percentage of invaded cells in the lower chamber was quantified and expressed based on untreated control cells. Images were taken using a 20× objective. (**E**) MDA-MB-231 cells were treated with UDP or MRS2578 for 12 h in the Matrigel invasion assay. The percentage of invaded cells in the lower chamber was quantified and expressed based on untreated control cells. Images were taken using a 20× objective. Three independent experiments were performed. Columns indicate the mean from three independent experiments with three duplicates; bars indicate the SE (*P < 0.05; **P < 0.01; ***P < 0.001 versus control).

To further confirm the function of P2Y_6_ in breast cancer cells, we detected the expression of P2Y_6_ in different cells lines by RT-PCR and Western blotting. As shown in Figure 2A and 2B, P2Y_6_ was highly expressed in many breast cancer cell lines, including MDA-MB-231, Hs578t, BT-549 and MCF-7. Thus, MDA-MB-231 cells were treated with UDP (10 μM and 100 μM) and MRS2578 (10 μM) to investigate cell migration and invasion using the scratch assay and Transwell assay. Interestingly, the migration of breast cancer cells was increased by UDP in a dose-dependent manner; this elevation was significantly inhibited by the selective P2Y_6_ antagonist MRS2578 in both the scratch (Figure 2C) and Transwell (Figure 2D) assays. Similarly, the invasion of MDA-MB-231 cells was also promoted by UDP and blocked by MRS2578 (Figure 2E). However, if we treated T47D cells (the expression of P2Y_6_ is very low) with UDP, the migration in both scratch and Transwell assays was little enhanced (Supplementary Figure S1A and S1B). These results show the inductive effect of UDP/P2Y_6_ in breast cancer cell migration and invasion.

### Knockdown of P2Y_6_ restricts breast cancer migration and invasion

**Figure 3:**
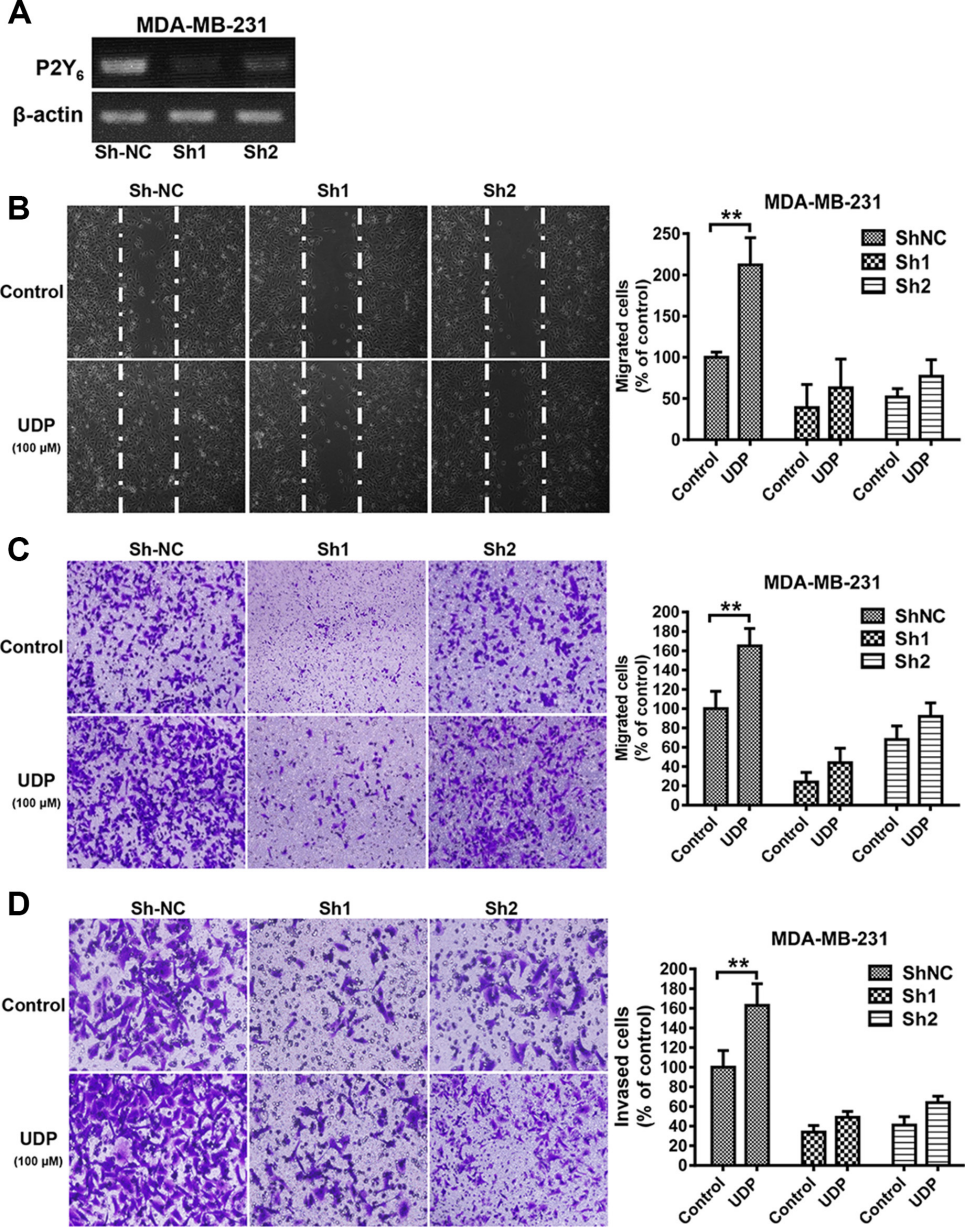
P2Y_6_ knock down reduces breast cancer cell metastasis (**A**) The expression of P2Y_6_ was detected by RT-PCR in control and P2Y_6_ knockdown MDA-MB-231 cells. (**B**) Control and P2Y_6_ knockdown MDA-MB-231 cells were scratched with a pipette tip and then treated with UDP for 24 h. Then, the migrated cells were fixed and counted. Images were taken using a 20× objective. (**C**) Control and P2Y_6_ knockdown MDA-MB-231 cells were incubated with UDP for 8 h in Transwell migration assays. The percent of migrated cells was quantified and expressed based on untreated control cells. Images were taken using a 20× objective. (**D**) For the Matrigel invasion assay, control and P2Y_6_ knockdown MDA-MB-231 cells were co-incubated with UDP 12 h. The percentage of invaded cells in the lower chamber was quantified and expressed based on untreated control cells. Images were taken using a 20× objective. Three independent experiments were performed. Columns indicate the mean from three independent experiments with three duplicates; bars indicate the SE (*P < 0.05; **P < 0.01; ***P < 0.001 versus control).

To further confirm the role of P2Y_6_ in breast cancer migration and invasion, we knocked down the expression of P2Y_6_ in MDA-MB-231 cells (Figure 3A). Under these conditions, not only the basal migration of P2Y_6_ knockdown cells obviously reduced, but UDP-induced migration was also eliminated in both the scratch (Figure 3B) and Transwell (Figure 3C) assays. Accordingly, similar data was also obtained from the cell invasion assay (Figure 3D). These results further confirm the important role of P2Y_6_ and its agonist UDP in breast cancer metastasis.

### Extracellular UDP is released by drug-damaged cells

**Figure 4:**
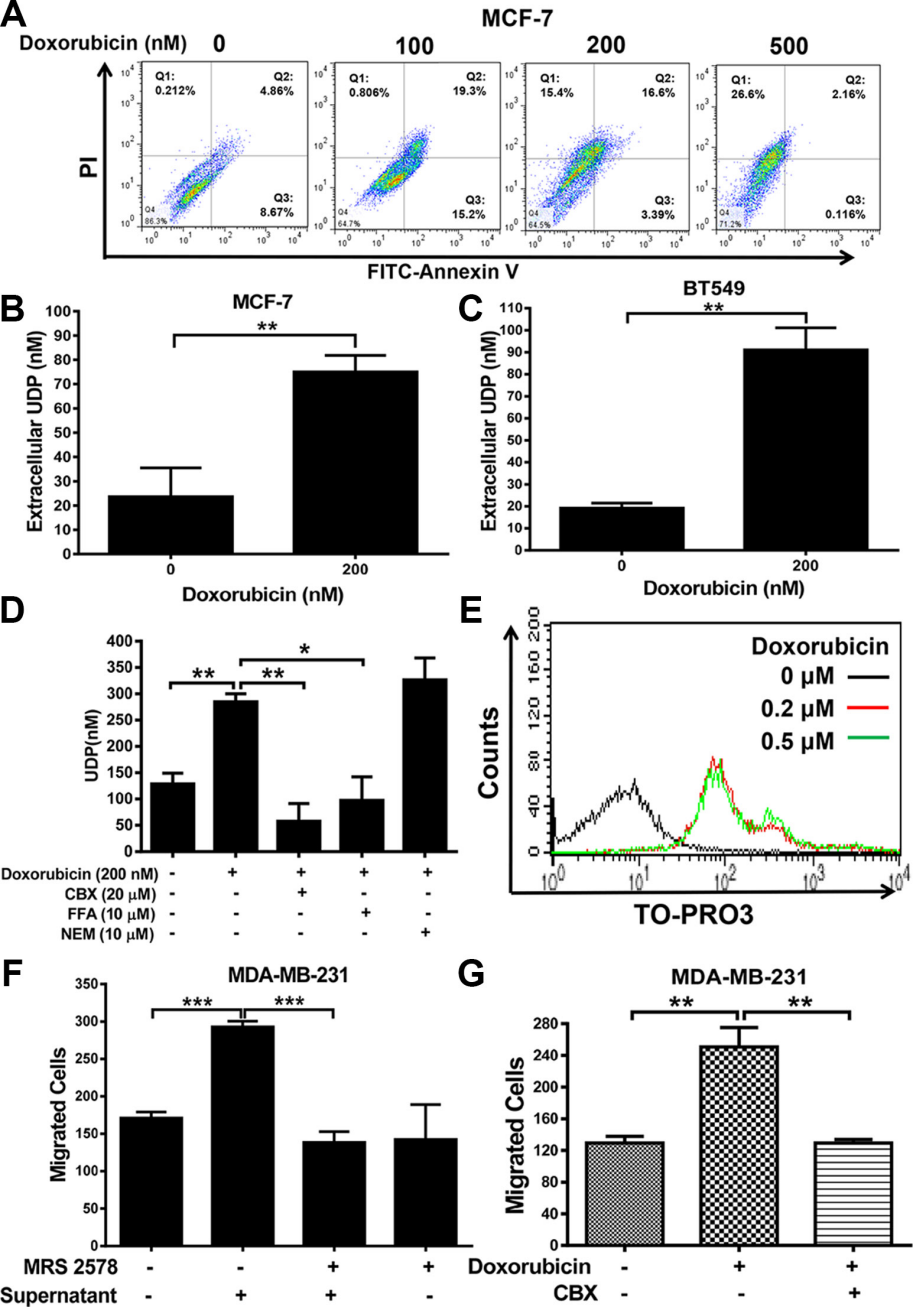
UDP released by doxorubicin induces apoptosis (**A**) MCF-7 cells were treated with different concentrations of doxorubicin for 24 h. Then, the cells were subjected to flow cytometric analysis of apoptosis. The results indicated that MCF-7 treated with doxorubicin was under apoptosis. (**B** and **C**) The supernatants of doxorubicin treated MCF-7 (B) and BT549 (C) cells were analyzed by the Transcreener UDP^2^ Assay. The concentration of UDP was calculated according to the standard curve. (**D**) Flow cytometric analysis of TO-PRO-3 uptake by MCF-7. Cells were stained with TO-PRO-3 after being treated with doxorubicin for 24 h. Then, the cells were subjected to a FACS assay to detect internalized TO-PRO-3. (**E**) MCF-7 cells were treated with doxorubicin and different channel inhibitors (CBX, FFA and NEM). Then, the concentration of UDP in the supernatant was detected by the Transcreener UDP^2^ Assay. (**F**) MDA-MB-231 cells were treated with the supernatants of doxorubicin or MRS2578 treated cells for 8 h in Transwell migration assays. The number of migrated cells was quantified and expressed based on untreated control cells. (**G**) MDA-MB-231 cells were pre-incubated with the supernatants of doxorubicin or CBX treated cells for 8 h in Transwell migration assays. The number of migrated cells was quantified and expressed based on untreated control cells. Three independent experiments were performed. Columns indicate the mean from three independent experiments with three duplicates; bars indicate the SE (*P < 0.05; **P < 0.01; ***P < 0.001 versus control).

The activation of purinergic receptors by specific extracellular nucleotides could initiate downstream signaling and associated functions. We have shown that P2Y_6_ is highly expressed in breast cancer tissues, but the mechanism by which the specific agonist UDP accumulates in the tumor microenvironment remained unclear. Therefore, we treated the cells with doxorubicin in a dose-dependent manner to set up a drug-induced cell damage or apoptosis model (Figure 4A). Consequently, significant accumulation of UDP in the supernatant of doxorubicin-treated cells was observed in both MCF-7 (Figure 4B) and BT-549 (Figure 4C) cells. Meanwhile, in order to explore why and how the danger signal is released from injured cells, we pre-treated cells with different inhibitors to nucleotide-release channels, including carbenoxolone (CBX, a nonspecific pannexin channel inhibitor), flufenamic acid (FFA, a gap junction inhibitor) and NEM (inhibits vesicular exocytosis) prior to drug treatment. As shown in Figure 3D, drug-triggered UDP release was inhibited by CBX and FFA, indicating that drug-induced UDP release occurs mainly through gap junction channels. Furthermore, the gap junction channel has been detected in doxorubicin-treated cells using the dye TO-PRO-3 [12], suggesting that the drug induced UDP release partially through pannexin-mediated gap junctions (Figure 4E). In addition, when MDA-MB-231 cells were treated with the supernatant from doxorubicin injured cells, the migration of MDA-MB-231 cells obviously increased; this degree of activation was reduced to normal levels by the selective P2Y_6_ inhibitor MRS2578 (Figure 4F) and the gap junction channel inhibitor CBX (Figure 4G). Taken together, our data demonstrate that drug damaged cells release UDP as a prometastatic signal to facilitate breast cancer metastasis, mainly through pannexin-mediated gap junctions.

### UDP/P2Y_6_ facilitate breast cancer cells metastasis *in vivo*

**Figure 5:**
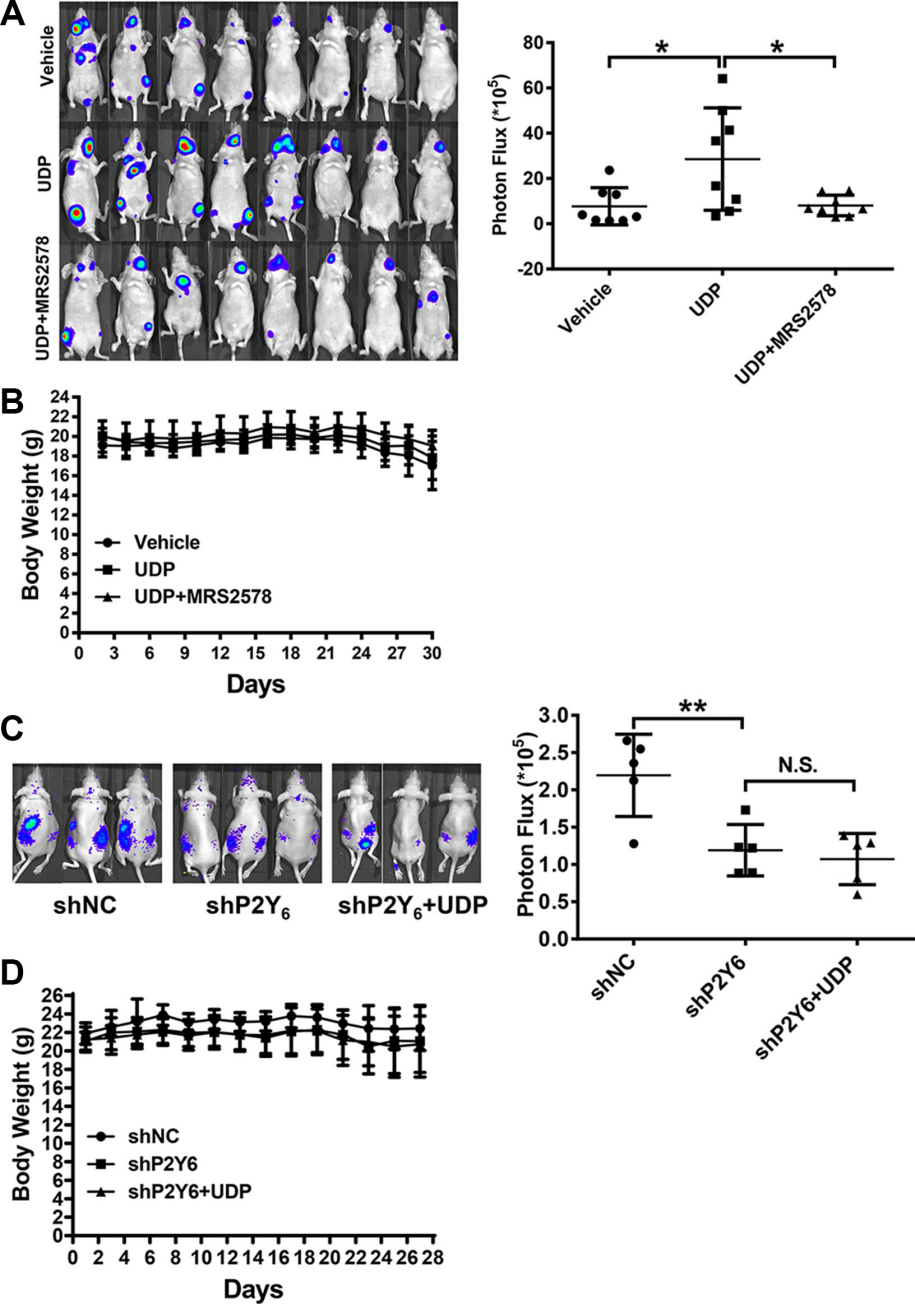
UDP/P2Y_6_ facilitates breast cancer cell metastasis *in vivo* (**A**) The breast cancer metastasis mouse model were set up by injecting 1 × 10^5^ firefly luciferase expressing MDA-MB-231 cells into nude mice. Mice were treated with 100 μM/day UDP (*n* = 8), 3 mg/kg/day MRS2578 (*n* = 8) or PBS vehicle (*n* = 8) subcutaneously. All animals were imaged for bioluminescence at day 30. Bioluminescence from the region of interest (ROI) was defined manually; the data are expressed as photon-flux (photons/s/cm^2^/steradian). ROI analysis of bioluminescence images was used to quantify breast cancer cell metastasis at day 30. (**B**) Meanwhile, the body weights of all mice were measured daily for 37 days after cancer cells were implanted. (**C**) 1 × 10^5^ firefly luciferase transfected control and P2Y_6_ knockdown MDA-MB-231 cells were injected into nude mice. Then, the mice were treated with 100 μM/day UDP (*n* = 5) or PBS vehicle (*n* = 5) subcutaneously. The bioluminescence image and ROI were set as described above. (**D**) Meanwhile, the body weight of all mice was measured daily after cancer cells were implanted.

For the *in vivo* breast cancer cell metastasis assay, Luc-MDA-MB-231 cells were injected and monitored using The Exnogen *In Vivo* Imaging System (IVIS, Alameda, CA. USA). As shown in Figure 5A, only a few metastasized cells were found near the jaw or hind limb in each mouse of the PBS treated group, whereas in the UDP treated group, the number of metastasized cells increased dramatically. This is consistent with the *in vitro* data, where UDP increased metastasis was also inhibited obviously by MRS2578. At the same time, the body weight of mice in each group was not significantly changed (Figure 5B). Subsequently, we knocked down the expression of P2Y_6_ in Luc-MDA-MB-231 cells to assess the function of P2Y_6_ in breast cancer metastasis. P2Y_6_ knockdown cells metastasized to a lesser extent than control cells, and UDP was also less effective in P2Y_6_ knockdown cells (Figure 5C). Again, there were few changes in the body weight of these mice (Figure 5D). These data further confirm the key role of UDP and P2Y_6_ in facilitating breast cancer cell metastasis.

### UDP/P2Y_6_ promotes MMP-9 expression and enzyme activity

**Figure 6:**
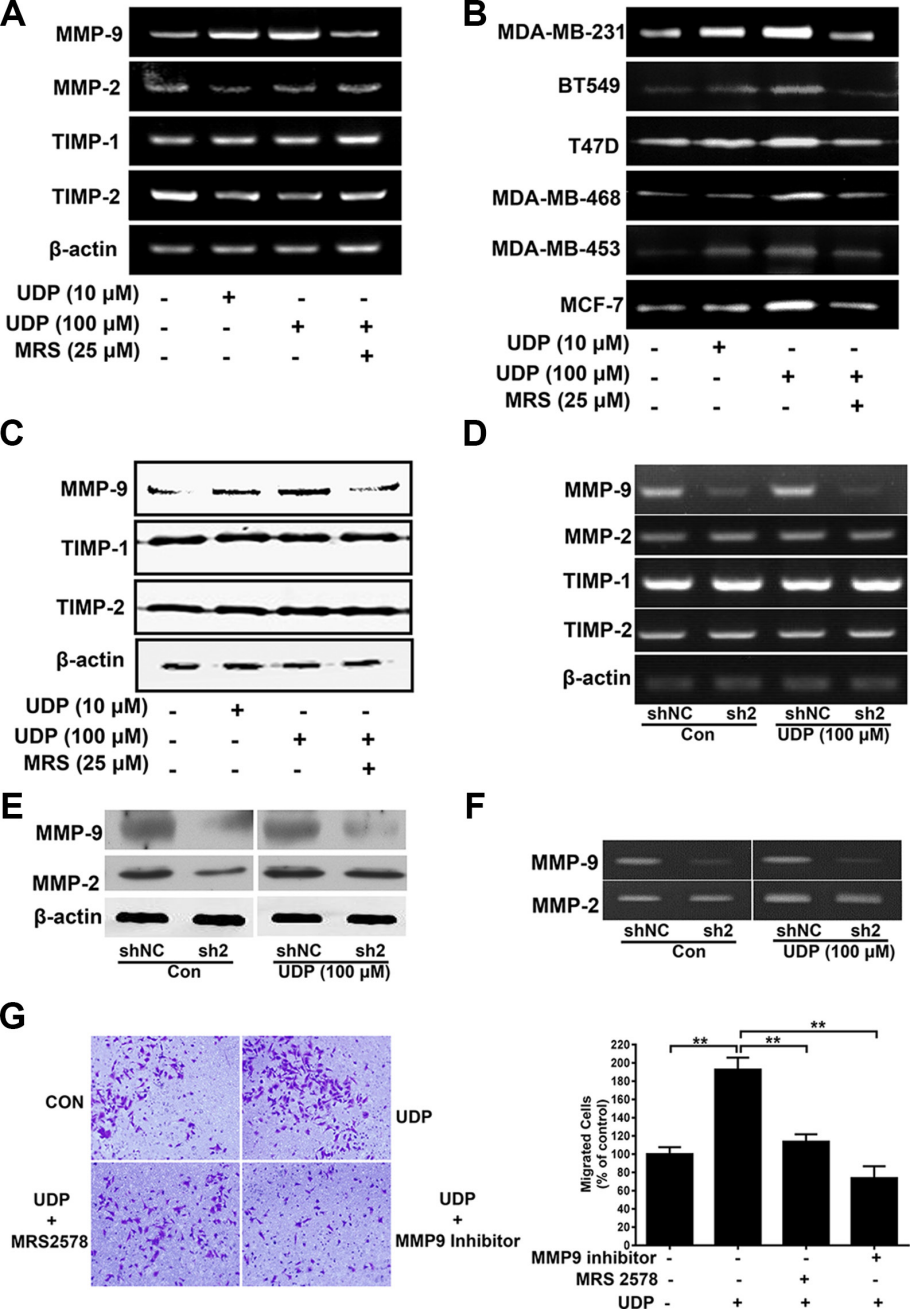
UDP/P2Y_6_ increases MMP-9 expression in breast cancer cells (**A**) RT-PCR analysis of MMPs mRNA expression in MDA-MB-231 cell. (**B**) Breast cancer cells were incubated with UDP for 24 h before pretreatment with MRS2578 for 1 h. Then, in the presence of different concentrations of UDP, the conditioned media were concentrated by 10-fold and analyzed by gelatin zymography. (**C**) Lysates from UDP and MRS2578 treated MDA-MB-231 cells were analyzed by Western blotting. (**D**) RT-PCR analysis of MMP mRNA expression in control and P2Y_6_ knockdown MDA-MB-231 cells. (**E**) Expression of MMP-9 in control and P2Y_6_ knockdown MDA-MB-231 cells was analyzed by Western blotting. (**F**) The conditioned media from control and P2Y_6_ knockdown MDA-MB-231 cells were concentrated by 10-fold and analyzed by gelatin zymography. (**G**) For the Matrigel invasion assay, cells were treated with UDP, MRS2578 or an MMP-9 inhibitor for 12 h. The percentage of invaded cells in the lower chamber was quantified and expressed based on untreated control cells. Images were taken using a 20× objective. Three independent experiments were performed. Columns indicate the mean from three independent experiments with three duplicates; bars indicate the SE (*P < 0.05; **P < 0.01; ***P < 0.001 versus control).

Matrix metalloproteinases (MMPs) are well-known ECM-degrading enzymes, and have essential roles in tumor progression, metastatic niche formation and inflammation in cancer [13]. Among them, MMP-2 (gelatinase-A) and MMP-9 (gelatinase-B) are mostly associated with tumor migration, invasion and metastasis in various cancers [14]. Interestingly, the mRNA expression (Figure 6A), enzyme activity (Figure 6B) and protein levels (Figure 6C) of MMP-9 in MDA-MB-231 cells were all increased by UDP in a dose-dependent manner, which could be inhibited by MRS2578 obviously. However, the mRNA expression of MMP-2, TIMP-1 and TIMP-2 was little affected. In addition, UDP increased MMP-9 enzyme activity, not only in MDA-MB-231 cells, but also in BT549, T47D, MDA-MB-468, MDA-MB-453 and MCF-7 cells, suggesting the extensive role of UDP/P2Y_6_ in breast cancer cell metastasis (Figure 6B). Similarly, when we knocked down the expression of P2Y_6_ in MDA-MB-231 cells, the mRNA expression (Figure 6D), protein level (Figure 6E) and enzyme activity (Figure 6F) of MMP-9 were all reduced. Similarly, the mRNA expression of MMP-2, TIMP-1 and TIMP-2 was not changed in P2Y_6_ knockdown cells. To further confirm the key role of MMP-9 in UDP-induced breast cancer cell metastasis, we treated the cells with UDP and MRS2578 or an MMP-9 inhibitor. Consequently, UDP-induced breast cancer cell invasion was significantly inhibited by both MRS2578 and the MMP-9 inhibitor (Figure 6G). These results imply MMP-9 may be the main driver of UDP/P2Y_6_ induced breast cancer metastasis.

### UDP/P2Y_6_ activates MAPKs and NF-κB associate signaling pathway

**Figure 7:**
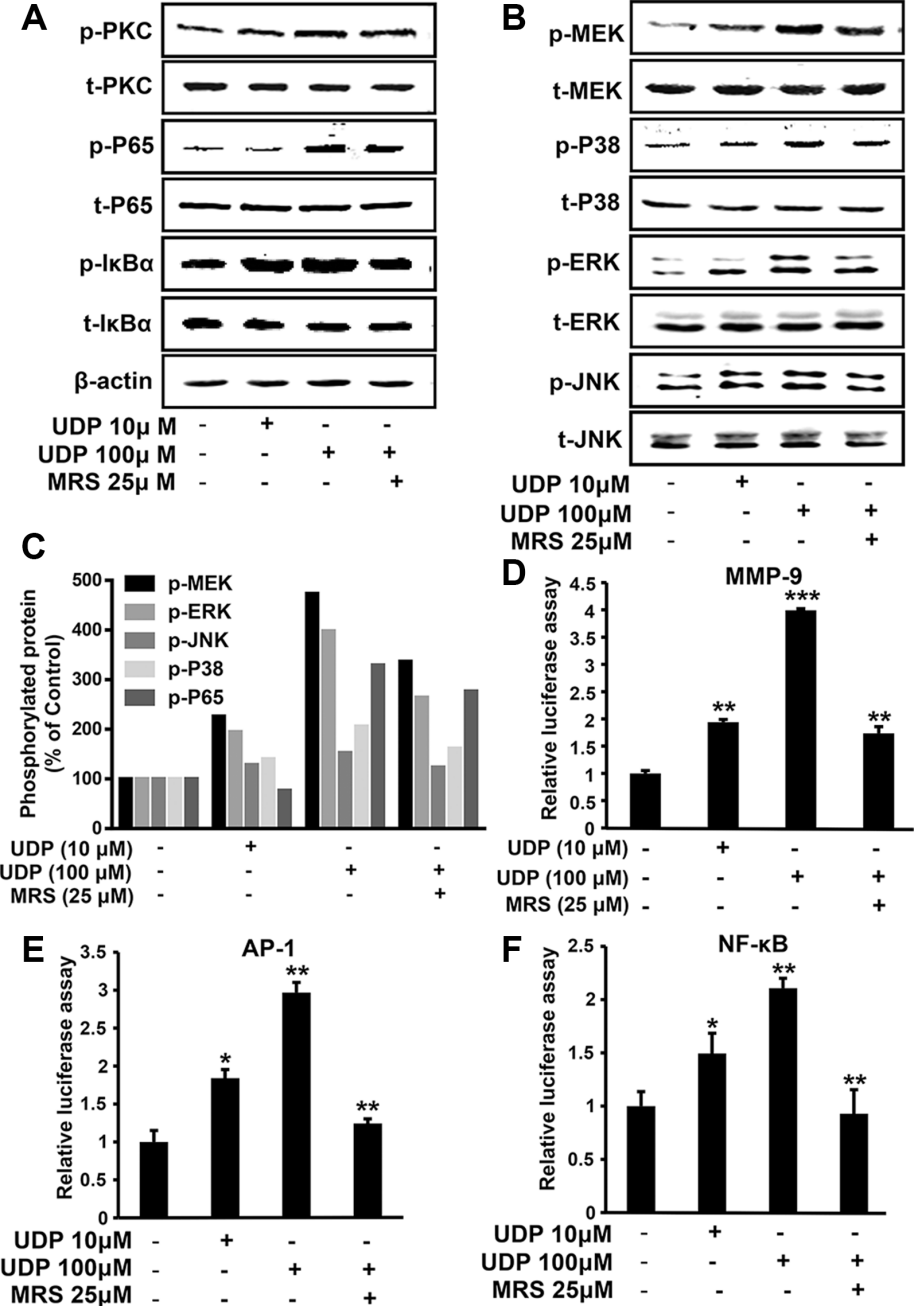
UDP/P2Y_6_ promotes breast cancer cell metastasis through NF-κB and MAPKs signaling (**A**–**C**) MDA-MB-231 cells were pretreated with MRS2578 for 1 h, then stimulated by UDP for 10 min, and the levels of phosphorylated protein kinases involved in NF-κB/MAPK associated signaling were determined (A and B) and quantified (C) by Western blotting with phospho-specific antibodies. Protein expression of β-actin in cell lysates was used as the internal standard. All the data was repeated at least 3 times. (**D**–**F**) For the luciferase assay, 293T cells were transfected with reporter vectors containing the binding sites for MMP-9 (A), AP-1 (D) and NF-κB (E), respectively. The cells were treated with MRS2578 and different concentrations of UDP for 12 h before luciferase activity was measured. Columns indicate the mean of luciferase activities calculated from three independent experiments; bars indicate the SE (*P < 0.05; **P < 0.01; ***P < 0.001 versus control).

Transcriptional induction of MMP-9 requires coordinated and cooperative activation of different transcription factors. As shown in Figure 7A, 7B and 7C, UDP stimulation resulted in a concentration-dependent increase in p38, MEK, ERK and p65 phosphorylation, whereas JNK was little affected, suggesting that P2Y_6_-induced MMP-9 expression occurs mainly through the phosphorylation of MAPKs and NF-κB. To determine whether UDP/P2Y_6_ receptor signaling affects the promoter activity of MMP-9, a luciferase reporter construct containing the MMP-9 promoter region was transiently transfected into HEK 293T cells. As shown in Figure 7D, MMP-9-luciferase activity was significantly activated by UDP, and this activation could be prevented by MRS2578. Then we assessed the influence of UDP/P2Y_6_ on AP-1 and NF-κB transcriptional activities by luciferase reporter assays. When treated with 100 μM UDP, the luciferase activity of AP-1 and NF-κB were both increased, but activation was reduced to normal levels following treatment with 25 μM MRS2578 (Figure 7E and 7F). These data suggest that P2Y_6_ facilitates breast cancer metastasis through the AP-1 and NF-κB signaling pathways.

## DISCUSSION

Recent studies have shown that the tumor microenvironment could be an important contributor to chemoresistance [15, 16]. Thus, a systematic dissection of the interactions between tumors and their microenvironment could be a novel way of underlying mechanisms of drug-induced resistance or metastasis [17, 18]. In this study, we found that extracellular UDP was released into the tumor microenvironment in response to doxorubicin treatment. In addition, released UDP activated highly expressed P2Y_6_ and facilitated breast cancer cell migration. Subsequently, when we treated these cell with MRS2578 or CBX (a specific inhibitor to pannexin channels), doxorubicin-induced breast cancer cell migration was greatly reduced, suggesting a key role of extracellular UDP in drug-induced resistance or metastasis. These results delineate an autonomous mechanism of drug treated breast cancer cells escaping from the tumor *in situ* through extracellular UDP and P2Y_6_ stimulation, indicating the great potential of purinergic signaling as a drug target in the prevention of breast cancer metastasis.

As the most comprehensive and coordinated tool in accelerating our understanding of the molecular basis of cancer through the application of genome analysis technologies, the Cancer Genome Atlas (TCGA) has accrued RNA-Seq-based transcriptome data from more than 4,000 cancer tissue samples across 13 cancer types [19]. Acquiring a deeper understanding of this information is important to identify therapeutic targets for cancer treatment [20]. In this study, we analyzed and compared P2Y_6_ expression in the transcriptomes of normal tissue samples and from 13 TCGA cancer types. Interestingly, very high amplification of P2Y_6_ was found in the BCCRC xenograft model, suggesting that P2Y_6_ is a potential target in breast cancer (Figure 1A). We then investigated the protein expression level of P2Y_6_ by immunohistochemistry assays in a tumor tissue array with 96 samples from 16 kinds of cancers. When compared with normal tissues, P2Y_6_ was very highly overexpressed in eight kinds of cancer, especially in breast cancer (Figure 1B and 1C). In addition, the expression of P2Y_6_ was also positively correlated with poor prognosis in breast cancer patients (Figure 1F). Taken together, the comprehensive and coordinated analysis of P2Y_6_ in a large number of clinical samples strengthens the correlation between P2Y_6_ and breast cancer and supports further study of P2Y_6_ expression in breast cancer and associated malignant characteristics.

**Figure 8:**
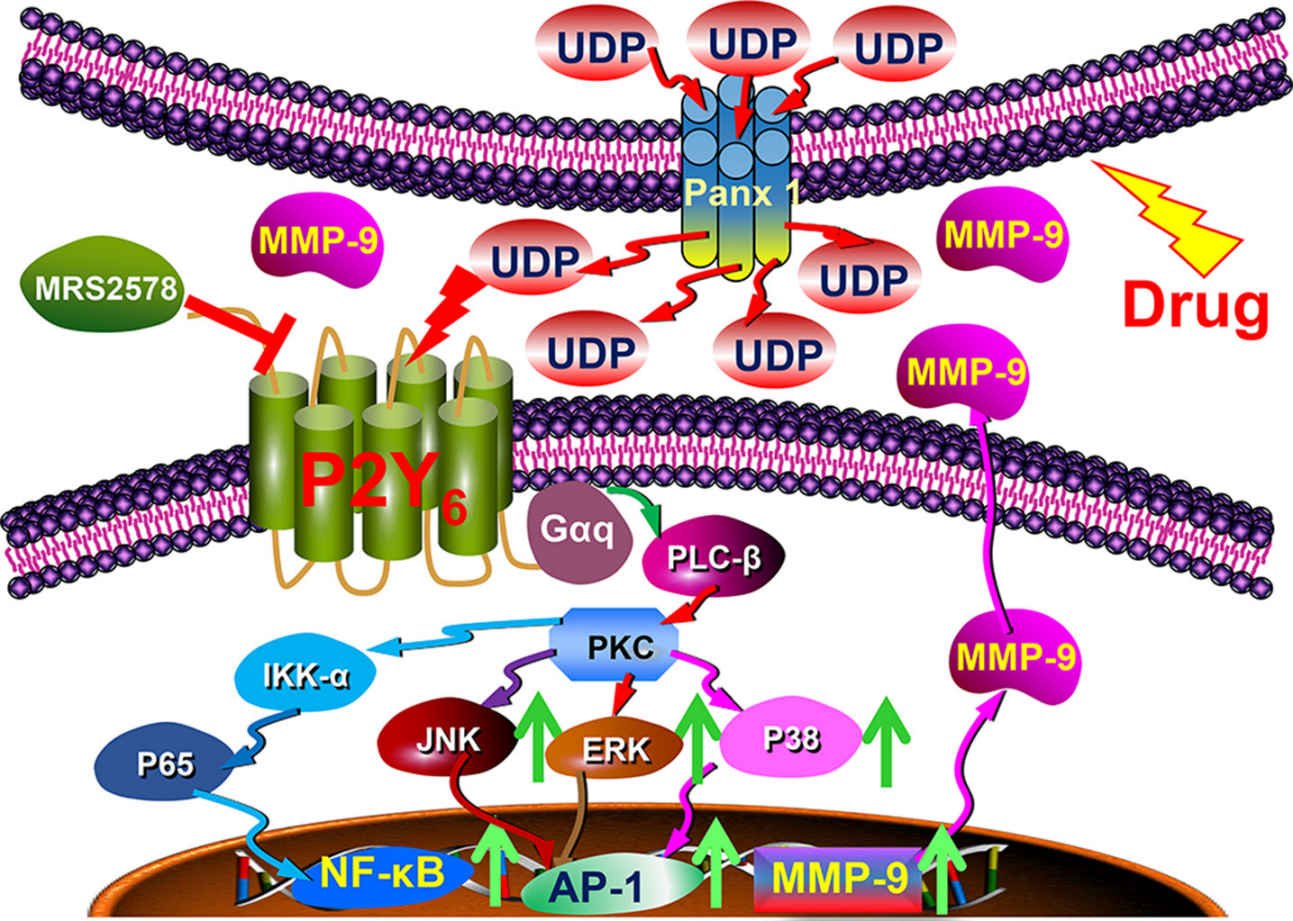
Schematic diagram showing the function and mechanism of UDP/P2Y_6_ facilitated breast cancer metastasis

The extracellular matrix and associated signals such as matrix metalloproteinases (MMPs), cytokine and growth factors play important roles in intercellular communications between tumor cells and stroma cells, and are also involved in tumor formation and progression [21–24]. Thus, MMPs were regarded as a good drug target for cancer therapy by nearly every pharmaceutical company [25, 26]. Interestingly, UDP release induces MMP-9 expression and enzyme activity in breast cancer cells through MAPK and NF-κB associated signaling pathways, which drive surrounding cancer cells to detach and escape from the primary site (Figure 6A–6C). In fact, a previous study has shown that cancer cells release UDP as a “find me” signal to recruit immune cells to clear dead or apoptotic cancer cells. Thus, our study broadens our understanding of extracellular UDP as an auto-regulator of tumor formation, progression and escape from chemotherapy, which indicates the potential therapeutic significance of UDP/P2Y_6_ associated signaling pathway in the prevention and control of breast cancer metastasis and drug resistance (Figure 8).

## MATERIALS AND METHODS

### Cells culture and reagents

The human breast carcinoma cell lines MDA-MB-231, BT549, T47D, MCF-7 and Hs578t, and the human kidney cell line HEK-293T were obtained from the China Type Culture Collection (Shanghai, China). MDA-MB-231 cells stably transfected with firefly luciferase (MDA-MB-231-Luc) were kindly provided by Dr. Qian Zhao at Shanghai Jiaotong University. The human breast cancer cell lines T47D and BT549 were cultured in RPMI-1640 supplemented with 10% FBS; Hs578t, MCF-7, MDA-MB-231 and HEK-293T were cultured in DMEM with 10% FBS. MDA-MB-231-luc cells were cultured in MEM containing 10% FBS, 1% 100 mM sodium pyruvate (Gibco) and 1% non-essential amino acids solution (Gibco). All the cell lines were maintained in an incubator with 5% CO_2_ at 37°C. Antibodies against MMP-9, TIMP-1/2, total and phosphorylated MAPK/ERK1/2, SAPK/c-Jun N-terminal Kinase (JNK) and p38 MAPK were purchased from Cell Signaling Technology (MA, USA). An antibody against P2Y_6_ (ab92504) was obtained from Abcam (MA, USA).

### Bioinformatics of P2Y_6_ expression across different cancer types

Copy-number alterations of P2Y_6_ across different cancer types were analyzed through the cBioPortal for Cancer Genomics (http://www.cbioportal.org/index.do) in 105 cancer genomics studies.

### Immunohistochemistry

The cancer tissue arrays (BR451, BR1008, BCN801, and BCN963) were purchased from US Biomax, Inc. Chips were first deparaffinized and rehydrated. Then chips were incubated in 3% hydrogen peroxide (dissolved in formaldehyde) for 10 min. For antigen retrieval, chips were incubated in 0.01 M sodium citrate (pH 6.0) at 100°C for 30 min. Non-specific binding sites were blocked by incubating chips in 1% bovine serum albumin (BSA) for 1 h. After that, chips were incubated at 4°C overnight with an antibody against P2Y_6_. The next day, chips were incubated with the secondary antibody (anti-rabbit IgG) for 1 h and then stained with HISTOSTAIN-Plus IHC Kit (DAB, Rabbit Primary) from Shanghai MRbiotech Co. Ltd. After being dehydrated, chips were observed and analyzed under a microscope (Leica DM4000B). Every sample on the microarray was imaged at three random locations. Moreover, every point was rated on a three-point scale (score 0, 1, 2, 3) in a blinded fashion.

### Kaplan-Meier plot analyses

Overall survival information was obtained from GEO (Affymetrix microarrays only), EGA and TCGA and presented as a Kaplan-Meier plot (http://kmplot.com/analysis/). A total of 1,117 patient samples were divided into two groups by comparing the median expression level of P2Y_6_, this was then set as the threshold for all samples to analyze the clinical expression of P2Y_6_. Two groups of samples were compared using the Kaplan-Meier survival plot. The hazard ratio, its 95% confidence intervals and logrank *P*-values were calculated and are listed in the figure.

### Reverse transcription-polymerase chain reaction (RT-PCR)

In the RT-PCR analysis, total RNA was extracted from treated cells. For the reverse transcription reaction, cDNA was synthesized from 1 μg of total RNA using Moloney murine leukemia virus reverse transcriptase (Promega). The PCR primers were: P2Y_6_ sense, 5′-CCGCTGAACATCTGTGTC-3′, P2Y_6_ antisense, 5′-AGAGCCATGCCATAGGGC-3′; MMP9 sense, 5′-TCCCTGGAGACCTGAGAACC-3′; MMP9 antisense, 5′-GGCAAGTCTTCCGAGTAGTTT-3′; MMP2 sense, 5′-GGAT GATGCCTTTGCTCG-3′; MMP2 antisense, 5′-ATCGGCGTTCCCATACTT-3′; TIMP-1 sense, 5′-GGGGACACCAGAAGTCAACCAGA-3′; TIMP-1antisense, 5′-CTTTTC AGAGCCTTGGAGGAGCT-3′; TIMP-2 sense, 5′-TGCAGCTGCTCCCCGGTGCAC-3′; TIMP-2 antisense, 5′-TTATGGGTCCTCGATGTCGAG-3′; β-actin sense, 5′-GCCATC GTCACCAACTGGGAC-3′; β-actin antisense, 5′-CGATTTCCCGCTCGGCCGTGG-3′. PCR products were analyzed by agarose gel electrophoresis and visualized by ethidium bromide staining.

### Apoptosis analysis

The cancer cell lines were starved for 12 h before treating with doxorubicin for 24 h. Then, cells were collected and stained for both Annexin V-FITC and propidium iodide (PI) according to the manufacturer's protocol using an Annexin V:FITC Apoptosis Detection Kit I (BD Pharmingen™, Catalog: 556547). Apoptosis was analyzed by flow cytometry on a BD FACSCalibur system according to the manufacturer's protocol.

### Generation of a P2Y_6_ stable knockdown cell line

The P2Y_6_ shRNAs (shRNA-1 forward oligo 5′-CCGGCATCTGTGTCATTACCCAGATCTCGAGATCTGGGTAATGACACAGATGTTTTTG-3′, reverse oligo: 5′-AATTCAAAA ACATCTGTGTCATTACCCAGATCTCGAGATCTGGGTAATGACACAGATG-3′; shRNA-2 forward oligo 5′-CCGGTGGTCCGCTTCCTCTTCTATGCTCGAGCATAGAAGAGGAAGCGGACCATTTTTG-3′, reverse oligo: 5′-AATTCAAAAATGGT CCGCTTCCTCTTCTATGCTCGAGCATAGAAGAGGAAGCGGACCA-3′) were cloned into the PLKO.1 plasmid flanked with Age I and EcoR I restriction sites. The P2Y_6_ shRNA positive insert plasmids, MD2.G and psPAX2 were transfected into HEK-293T together. After 8 h, the medium was replaced with fresh DMEM containing 10% FBS. Then, the HEK293T cells were incubated for 24 h. The medium was harvested and filtered through a 0.45 μm filter. The media containing the virus was then used to infect BT549 cells for 8 h. Positive cells were selected based on puromycin resistance and analyzed by RT-PCR.

### Cell invasion and migration assay

Invasion and migration of breast cancer cells were assessed by performing Transwell and scratch assays. The Transwell assay is conducted by counting cancer cells that have migrated across the membrane filter (8 μm pore size, Millipore Corporation) of the Transwell insert in a given period of time. For the Transwell migration assay, the lower well of the chamber was filled with migration-inducing medium (10% FBS) and the upper wells were seeded with 5 × 10^4^ MDA-MB-231 cells per well with different concentrations of UDP. The cells were pretreated with MRS2578 for 1 h if required. After 8 h, upper wells were fixed with 100% methanol for 20 min at room temperature and then stained with crystal violet for 20 min. Cells in the upper chamber that did not migrate were removed. Images were taken using a microscope (OLYMPUS IX71) and migrated cells were evaluated by manual counting. The percentage inhibition of cell migration was quantified and expressed based on untreated control wells. For the Transwell invasion assay, the upper well of chamber was coated with 20 μl of Matrigel (1.5 mg/ml), while the lower well of the chamber was filled with medium containing 10% FBS. Cells (1 × 10^5^ cells per well) were seeded in the upper well and treated with UDP and MRS2578 for 12 h. Then, the cells were observed and quantified as above.

### Scratch assay

In preparation for the scratch assay, breast cancer cells were plated in 12-well plates and starved for 12 h. Then, cancer cells were scraped using pipette tips and washed with PBS. Cancer cells were pretreated with MRS2578 for 1 h if required; cells were then treated with different concentrations of UDP and were allowed to migrate for 24 h in FBS-free medium. Images were taken using a microscope (OLYMPUS IX71) and migrated cells were quantified by manual counting. The percentage inhibition of migrated cells was quantified and expressed based on untreated control wells.

### UDP release analysis

The release of UDP was detected using the Transcreener UDP^2^ Assay (BellBrook Labs) through a fluorescence polarization readout. A standard curve was prepared by standard UDP solution before analysis. Cancer cells were cultured in DMEM without phenol red (Gibco) and starved for 12 h in advance. After cancer cells were treated with doxorubicin, the supernatant of cells was removed and incubated on ice. According to the manufacturer's protocol, a 15 μl mixture of reagents for every well was prepared beforehand, to which 5 μl of cancer cell supernatant was added. The plate was incubated for 1.5 h, then the fluorescence polarization of each well was analyzed by a FlexStation 3 (Molecular Devices). The concentration of UDP was calculated using the standard curve.

### TO-PRO-3 uptake analysis

The cancer cell lines were starved for 12 h before treatment with doxorubicin for 24 h. Cells were collected in tubes and stained with 600 nM TO-PRO-3 (Thermo) for 10 min. Uptake of TO-PRO-3 was analyzed by flow cytometry on a BD FACSCalibur system according to the manufacturer's protocol.

### Western blot

Cells were plated onto 6-well plates and starved overnight, then whole-cell extracts were prepared in RIPA buffer (20 mM Tris, 2.5 mM EDTA, 1% Triton X-100, 1% deoxycholate, 0.1% SDS, 40 mM NaF, 10 mM Na_4_P_2_O_7_ and 1 mM PMSF). All samples were separated by sodium dodecyl sulfate polyacrylamide gel electrophoresis to assess the expression of P2Y_6_ and proteins associated with cell invasion and migration.

### *In vivo* metastasis animal model

For in the vivo metastasis assay, MDA-MB-231 cells expressing firefly luciferase were used. Briefly, 1 × 10^5^ cancer cells were injected into the left ventricle of 5-week-old female nude mice (Bikai, Shanghai, China). The next day, mice were injected intraperitoneally with UDP (100 μM), MRS2578 3 mg/kg or the same volume of PBS vehicle (containing 1% DMSO) every day for about 30 days. Finally, the mice were injected with 150 mg/kg D-luciferin (potassium salt; Promega) and taken for bioluminescence imaging using the Exnogen *In Vivo* Imaging System (IVIS, Alameda, CA.USA). Bioluminescence from the ROI was defined manually, and data are expressed as photon flux (photons/s/cm^2^/steradian). Background photon flux was defined from an ROI drawn over a mouse that was not given an injection of luciferin. All mice were culled at day 40 to reduce suffering from metastasized cancer cells; most PBS treated mice had died before this time point. All animal procedures were approved by the East China Normal University Center for Animal Research and conformed to the regulations drafted by the Association for Assessment and Accreditation of Laboratory Animal Care in Shanghai.

### Cell viability assay

Cells were seeded onto 96-well plates, then treated with 10 μM or 100 μM UDP or together with MRS2578 25 μM for 24 h. Every well of plate received 20 μl of MTS solution (Promega) and was incubated for 2.5 h at 37°C. The OD value was measured at 490 nm using a microplate reader (Molecular Devices).

### Gelatin substrate gel zymography

For the gelatin zymography assay, breast cancer cells were plated onto 6-well plates and incubated until they reached 80% confluence, then the medium was changed to fresh serum-free medium containing different concentrations of UDP and MRS2578. After 24 h, the supernatants were collected and concentrated. The resultant supernatants were subjected to SDS-PAGE on 8% polyacrylamide gels that were copolymerized with 1 mg/ml of gelatin. After the electrophoresis runs, the gels were washed several times with 2.5% Triton X-100 for 1 h at 4°C to remove the sodium dodecyl sulfate and incubated for 12 h at 37°C in a buffer containing 50 mM Tris-base, 200 mM NaCl, 10 mM CaCl_2_ and 1 μM ZnCl_2_. The gels were stained with 0.25% Coomassie Brilliant Blue R250 (Bio-Rad, Hercules, CA) for 1 h, and then destained for 1 h in a solution of acetic acid and methanol. Proteolytic activity was evidenced as clear bands against the blue background of the stained gelatin.

### Luciferase reporter assay

Luciferase reporter assay was carried out by using HEK-293T cells. Cells were seeded into 10 cm cell culture dish. When achieved 70% confluence, 8 μg pAP-1-luciferase vector, 1 μg of MMP-9 promoter-luciferase reporter constructs or pNF-κB-luciferase vector (are all pGL3 based) were transfected into cells using lipofectamine 2000 (Invitrogen) [27]. After transfection, cells were replated into 24-well plate and treated with different concentrations of UDP and MRS2578 for 12 h. Finally, the cells were washed and lysed with luciferase lysis buffer for the luciferase assay. Renilla luciferase reporter was used as a transfection efficiency control and the relative luciferase activities were measured against renilla luciferase activities following the manufacturer's protocol (Luciferase Assay System, Promega) with a Victor 3 microplate reader (PerkinElmer).

### Statistical analysis

All data were imported into GraphPad Prism 6.0 software and are presented as mean ± SEM. Statistical significance was assessed by a two-tailed unpaired Student's *t*-test or one-way variance (ANOVA), where appropriate, with SPSS 20.0 software (IBM). Statistical significance was at *p* < 0.05.

## SUPPLEMENTARY FIGURE


