# Supplementary material for: Chemotherapy-induced uridine diphosphate release promotes breast cancer metastasis through P2Y6 activation
Source: Oncotarget. 2016 Apr 9;7(20):29036–50. doi: 10.18632/oncotarget.8664 (PMC5045376; doi:10.18632/oncotarget.8664)
Supplement: Supplementary file 1 [file oncotarget-07-29036-s001.pdf]

Chemotherapy-induced uridine diphosphate release promotes breast cancer metastasis through P2Y$_6$ activation

Supplementary Materials

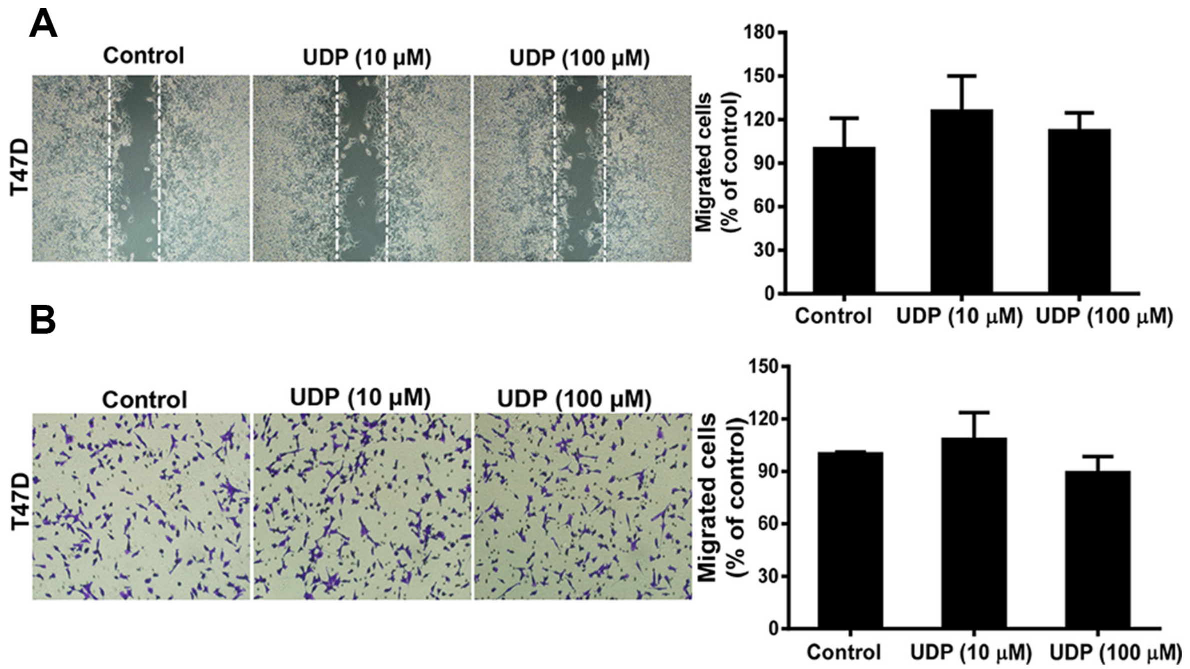

Supplementary Figure S1: The migration of T47D cells is little influenced by UDP. (**A**) T47D cells were scratched with a pipette tip and then treated with UDP for 24 h. Then, the migrated cells were fixed and counted. Images were taken using a 20× objective. (**B**) T47D cells were pre-incubated with UDP for 8 h in Transwell migration assay. Cells on the bottom side of the filter were fixed, stained and counted. The percentage of invaded cells in the lower chamber was quantified and expressed based on untreated control cells. Images were taken using a 20× objective.
